# NeuropsychopharmARCology: Shaping Neuroplasticity through Arc/Arg3.1 Modulation

**DOI:** 10.2174/011570159X338335240903075655

**Published:** 2024-10-25

**Authors:** Francesca Mottarlini, Lucia Caffino, Fabio Fumagalli, Francesca Calabrese, Paola Brivio

**Affiliations:** 1 Department of Pharmacological and Biomolecular Sciences ‘Rodolfo Paoletti’, Università degli Studi di Milano, Via Balzaretti 9, 20133 Milano, Italy

**Keywords:** Arc/Arg3.1, immediate early genes, antidepressants, antipsychotics, psychostimulants, CNS disorders

## Abstract

Activity-regulated cytoskeleton-associated protein (aka activity-regulated gene Arg3.1) belongs to the effector gene family of the immediate early genes. This family encodes effector proteins, which act directly on cellular homeostasis and function. Arc/Arg3.1 is localized at dendritic processes, allowing the protein local synthesis on demand, and it is considered a reliable index of activity-dependent synaptic changes. Evidence also exists showing the critical role of Arc/Arg3.1 in memory processes. The high sensitivity to changes in neuronal activity, its specific localization as well as its involvement in long-term synaptic plasticity indeed make this effector gene a potential, critical target of the action of psychotropic drugs. In this review, we focus on antipsychotic and antidepressant drugs as well as on psychostimulants, which belong to the category of drugs of abuse but can also be used as drugs for specific disorders of the central nervous system (*i.e*., Attention Deficit Hyperactivity Disorder). It is demonstrated that psychotropic drugs with different mechanisms of action converge on Arc/Arg3.1, providing a means whereby Arc/Arg3.1 synaptic modulation may contribute to their therapeutic activity. The potential translational implications for different neuropsychiatric conditions are also discussed, recognizing that the treatment of these disorders is indeed complex and involves the simultaneous regulation of several dysfunctional mechanisms.

## 1 INTRODUCTION

Immediate early genes (IEGs) are divided into two separate classes, *i.e*., the classical transcription factors, whose function is to regulate the expression of other genes, and the effector genes, which influence cellular homeostasis and function [1, 2]. Activity-regulated cytoskeleton-associated protein (Arc; aka activity-regulated gene Arg3.1) belongs to the effector gene class [1]. The most interesting feature of Arc/Arg3.1 is that it is rapidly induced by neuronal activity; accordingly, Arc/Arg3.1 mRNA moves into activated dendrites, where it is translated [3, 4]. The mechanism responsible for this movement was shown to be dependent on actin polymerization and ERK phosphorylation [5] as well as on glutamatergic NMDA receptor activation [6]. Once accumulated at synapses, synaptic activity causes mRNA decay, removing Arc/Arg3.1 mRNA from inactive dendrites [7]. Another Arc/Arg3.1 mRNA degradation mechanism occurs through Ube3A, an E3 ubiquitin ligase induced by experience-driven neuronal activity [8]. While there is no question about the fact that Arc/Arg3.1 mRNA is locally translated in dendrites, recent data have demonstrated that it is not only found in active synapses but also in inactive or less active synapses [9]. This unexpected localization may play a role during the late phase of long-term synaptic plasticity, during homeostatic plasticity and synaptic scaling [10-12]. Interestingly, Arc/Arg3.1 has been observed in glia [13, 14]. Despite Arc/Arg3.1 is generally considered a post-synaptic protein, evidence exists that it can also be presynaptic since it has been found to colocalize with presynaptic proteins [15, 16]. In addition, Arc/Arg3.1 has been observed not only in dendrites but also at high levels in the nucleus [17, 18], adding complexity as well as specificity to the modulation of Arc/Arg3.1 and opening the possibility that Arc/Arg3.1 may have different roles depending on its subcellular localization.

Arc/Arg3.1 appears to play a role, both at preclinical and clinical levels, in the central nervous system (CNS) disorders such as Alzheimer’s disease, schizophrenia, and depression [19-23]. Recent data have pointed out a role for Arc/Arg3.1 in drug abuse, and this is in line with the evidence that at least the acute modulation of this effector gene is extremely sensitive to the stimulation of the main dopaminergic receptors D_1_ (D_1_R) and D_2_ (D_2_R) [24], known to be critical in the regulation of reward. It is interesting to underline that Alzheimer’s disease, psychiatric disorders, and substance abuse share the presence of cognitive deficit as a common denominator and, therefore, considering the role of Arc/Arg3.1 in synaptic plasticity, which is crucial for cognitive processes, it is not surprising that Arc/Arg3.1 is implicated in the dysfunctions that characterize these disorders.

**Fig. (1):**
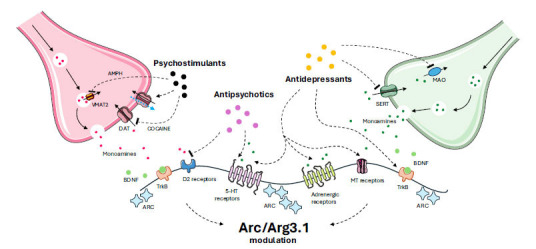
Key mechanisms of action for antidepressants, antipsychotic drugs, and psychostimulants. Among their multi-receptor targeting, antipsychotic drugs mainly act by blocking the D_2_ receptors, whereas the primary mechanism of antidepressant drugs is the blockage of the serotonin transporter SERT. However, their mechanism of action is more complex, so for graphical clarity, we represented the modulation of the drug classes on the different receptors with dashed arrows.

In this review, an overview of the role of Arc/Arg3.1 in synaptic plasticity will be provided. Next, the modulation of Arc/Arg3.1 expression after exposure to antipsychotic (AP) and antidepressant (AD) drugs, both chronic and acute, will be reviewed to be able to separate the effects set in motion by regulation of neurotransmitter receptors (acute effects) from those that occur as a result of neuroadaptive changes (long-lasting effects). The role of Arc/Arg3.1 in the action of drugs of abuse will be addressed, with a primary focus on changes in neuroplasticity (Fig. **1**). The review will be concluded with an interpretation and discussion of the main findings available. To make reading easier, all the studies cited in the text are summarized in Table **1** to quickly have the different drugs at hand, their respective dosages, and the brain regions being studied.

## 2 THE CRITICAL ROLE OF ARC/ARG3.1 IN SYNAPTIC PLASTICITY

Synaptic plasticity can be defined as a way of storing information to be used by the synapse on demand. In the presence of a stimulus, neurons change their strength and connectivity through the rapid response set in motion by IEGs. Among the different IEGs, Arc/Arg3.1 regulates long-term synaptic plasticity, including long term potentiation (LTP) and long-term depression (LTD), suggesting its requirement for both strengthening and weakening of the synapses, as well as homeostatic scaling, indicating its fundamental role in adjusting synaptic strength to prolonged changes in neuronal activity. Despite Arc/Arg3.1 exerting these actions through different processes [25] and cell-type specific mechanisms, information in this regard is still limited. Recent findings hypothesize that Arc/Arg3.1 functions tightly depend on different oligomeric forms of this effector gene, with monomer/dimer functioning in LTP, whereas tetramer works for LTD [26]. In addition, evidence exists that Arc/Arg3.1 is required for BDNF-dependent LTP in the dentate gyrus of the hippocampus [27, 28] and mGluR1-induced LTD in the CA1 hippocampal subregion [29]. In mammals, Arc/Arg3.1 is predominantly expressed in excitatory glutamatergic neurons of the CNS, and the different forms of synaptic plasticity regulated by increased Arc/Arg3.1 expression mainly take place in the postsynaptic density of excitatory glutamatergic neurons. Arc/Arg3.1 has been shown to regulate endocytosis and trafficking as well as homeostatic scaling of AMPA receptors in memory consolidation [10-12] but not in learning [30-34]. Notably, it is also a key factor in modulating structural plasticity, as evidenced by its role in regulating spine morphology and actin dynamics in dendritic spines [35].

Recent studies showed that Arc/Arg3.1 has retrovirus properties and evolved from ancient retrotransposons. In detail, Arc/Arg3.1 has been demonstrated to encode a protein that forms virus-like capsids, which mediate intercellular transfer of Arc/Arg3.1 mRNA in neurons, presumably to control synaptic function and plasticity [36]. In addition, recent work from Avallone and colleagues (2023) has provided elegant evidence for interneuronal *in vivo* transfer of Arc/Arg3.1 in the brain [16], a possibility previously suggested *in vitro* [36]. Accordingly, intercellular mRNA transport in secreted virus-like particles represents a new feature of Arc/Arg3.1 activities.

Using the rodent visual cortex as a model, Arc/Arg3.1 has also been shown to play a role in experience-dependent plasticity. In fact, in this brain region, which is crucial for visual information processing, brief monocular deprivation during the critical period leads to a marked plasticity in juvenile rodents that is abrogated by Arc/Arg3.1 removal [37]. Notably, these authors also showed that, by overexpressing Arc/Arg3.1, such plasticity is extended in the visual cortex, suggesting that the availability of Arc/Arg3.1 is critical for adult plasticity. In addition, since it is known that LTD mechanisms mediate eye deprivation [38], these lines of evidence point to Arc/Arg 3.1 in the long-term processes subserving experience-dependent plasticity. Further, it is interesting to point out that the antidepressant fluoxetine, known to promote Arc/Arg3.1 expression, restores neuronal plasticity in the adult visual system of the rat [39], strengthening the link between psychotropic drugs and Arc/Arg3.1-mediated induction of experience-dependent plasticity.

Arc/Arg3.1 also plays an important role in synaptic changes underlying plastic modifications necessary for long-term memory formation, including impaired long-term novel object recognition memory, contextual and cued fear conditioning, as well as reduced conditioned taste aversion [33]. Of note, the selective blockage of hippocampal Arc/Arg3.1 expression mimicked the same profile of behavioral deficit just mentioned [34, 40, 41]. It is interesting to point out that the conditional removal of Arc/Arg3.1 impairs spatial learning early in life but not in adulthood, suggesting a differential role of Arc/Arg3.1 that depends upon brain maturation [32]. Notably, recent evidence suggests that reduced Arc/Arg3.1 expression accelerates the process of forgetting in aged animals [42]. Interestingly, Arc/Arg3.1 appears to be critical also for aversive memories since knockout or knockdown of Arc/Arg3.1 in the lateral amygdala causes a deficit in fear conditioning memories [43].

Taken together, these data indicate that Arc/Arg3.1 couples change in synaptic activity with the expression of structural and functional markers of plasticity, pointing to this effector molecule as a potential target for pharmacological treatments aimed at restoring compromised neuroplasticity that occurs in several disorders of the CNS.

## 3 EFFECT OF ANTIPSYCHOTIC DRUGS ON ARC/ARG3.1 MODULATION

### 3.1 Acute Exposure

Recent studies show the involvement of genetic variations in schizophrenic patients on synaptic proteins found to interact with Arc/Arg3.1 [44-47]. Almost concomitantly, a reduction of Arc/Arg3.1 expression was found in the prefrontal cortex (PFC) of schizophrenic patients [48], and associations were reported between the single nucleotide polymorphisms (SNP) of the Arc/Arg3.1 gene and schizophrenia [49]. In this study, the authors showed an SNP in an Arc/Arg3.1 intron; such SNP is significant in USA populations of European and African descent, suggesting that Arc/Arg3.1 could be involved in schizophrenia risk; however, no function has been assigned to this SNP so far [49]. In addition, Marshall and collaborators (2017) found several deletions significantly enriched in schizophrenic patients among synaptic proteins interacting with Arc/Arg3.1 [50]. Such lines of evidence suggest an association between Arc/Arg3.1 and schizophrenia, but the potential causal implication is still far from being proven.

In rodents, reductions in Arc/Arg3.1 expression have been found in animal models of schizophrenia [51, 52]. Gao and associates (2018) have demonstrated that genetic deletion of Arc/Arg3.1 does not cause schizophrenia-like behavior, thus questioning the role of Arc/Arg3.1 in this disorder [32]. Conversely, Managò and coworkers (2016) showed that mice with the deletion of Arc/Arg3.1 develop schizophrenia-relevant behavioral phenotypes (prepulse inhibition deficit, cognitive dysfunctions, impaired social behaviors) [53]. The reason for such discrepancy might rely on important differences in how the knockouts were obtained. Interestingly, the authors showed that local injection of a D_1_R agonist in the PFC or application of a D_2_R antagonist in the ventral striatum rescued cognitive and psychomotor dysfunctions. The effects of these dopamine-related drugs fit nicely with previous evidence showing that Arc/Arg3.1 expression is under the tight control of D_1_R and D_2_R receptors; in fact, D_1_R agonists up-regulate Arc/Arg3.1 mRNA levels in the striatum and PFC whereas the blockage of these receptors by SCH 23390 down-regulates Arc/Arg3.1 mRNA levels in both brain regions [24]; conversely, the D_2_R/D_3_R agonist quinpirole down-regulates Arc/Arg3.1 mRNA levels in the striatum with no effects in the PFC and the D_2_R antagonist raclopride increases Arc/Arg3.1 mRNA levels in rat striatum while reducing its gene expression in the PFC [54]. Interestingly, the modulation of Arc/Arg3.1 by these drugs seems to have functional relevance, as quinpirole caused synaptic rearrangements in cortical neuron cultures by reducing spine branches and length [55]. Interestingly, it has been shown that Arc/Arg3.1 also plays a critical role in cognitive, social, and arousal processes through mechanisms that involve not only dopamine but also glutamate [56], suggesting that individual variability in Arc/Arg3.1 genetics may fuel precision medicine as well as prevention approaches in altered cognitive and social processes in schizophrenia.

Taken together, these results point to regionally distinct dopaminergic mechanisms in the regulation of Arc/Arg3.1, which heavily depends on the relative expression of D_1_R or D_2_R. However, it is well established that Arc/Arg3.1 up-regulation relies also on the activation of glutamate NMDA receptors. In addition, evidence exists that dopamine, primarily through D_2_R, regulates glutamate release. The striatal increase of Arc/Arg3.1 mRNA levels caused by D_2_R antagonists may depend on cortico-striatal glutamate release caused by D_2_R, primarily on striatal medium spiny neurons [57, 58]. Thus, it is hypothesized that the blockage of NMDA receptors would indirectly up-regulate the activity of striatal NMDA receptors, causing up-regulation of Arc/Arg3.1 mRNA levels; conversely, the agonism at D_2_R would diminish glutamate release onto medium spiny neurons, reducing NMDA receptor activation and thereby reducing Arc/Arg3.1 mRNA levels. At variance from these D_2_R-dependent mechanisms, the effects of D_1_R agonists are presumably driven by the localization of these receptors on medium spiny neurons, which up-regulated NMDA responses, thus increasing Arc/Arg3.1 mRNA levels, whereas an opposite mechanism is suggested for D_1_R antagonists [59, 60]. A different situation has been observed when dopaminergic stabilizers are administered (compounds that modulate dopamine transmission in a more balanced way, *i.e*., exhibiting D_2_R antagonism but a more attenuated effect on dopamine-dependent psychomotor functions) [61]. Notably, the dopaminergic stabilizers pridopidine and ordopidine dose-dependently increased Arc/Arg3.1 mRNA levels in both the striatum and frontal cortex [62]. The potential explanation of this effect relies on the fact that such stabilizers may increase the activity of cortical pyramidal cells, as suggested by the evidence that the selective D_1_R antagonist SCH 23390 attenuates such activity [63]. Thus, the stimulation of D_1_R should then increase NMDA receptor activity and enhance Arc/Arg3.1 cortical mRNA levels [64, 65].

If we shift our attention from agonists/antagonists, with extreme selectivity toward specific dopamine receptors, to AP drugs used in therapy, which, by definition, are not selective, we can discriminate between acute treatments (involving analysis of time dependence and dose-response) or chronic treatments, which imply the repeated exposure with the consequent neuroadaptations. Following this line of reasoning, Arc/Arg3.1 may bridge together drug-induced neuronal activity with drug-induced neuroplasticity. It is well established that the modulation of IEGs after acute AP injection is due to the activation of rapid synaptic mechanisms. We have demonstrated that the first-generation antipsychotic (FGA) haloperidol up-regulates Arc/Arg3.1 mRNA striatal levels at the three different time points investigated (30 minutes, 1 and 2 hours) [54]. It is interesting to point out that the second-generation antipsychotic (SGA) olanzapine increases striatal Arc/Arg3.1 mRNA levels [54], an effect that, however, vanishes 1 hour following the injection; this is likely due to the lower affinity blockage exerted by olanzapine, which indeed blocks D_2_R at a lower affinity than haloperidol [65], in line with the lower k_off_ of haloperidol [66]. Interestingly, another SGA, quetiapine, exhibiting a D_2_R affinity lower than olanzapine, does not alter striatal Arc/Arg3.1 mRNA levels [54], suggesting that the different affinity of APs for D_2_R may predict the effect on striatal Arc/Arg3.1 mRNA levels. Instead, in the frontal cortex, we observed an opposite expression profile to that seen in the striatum but similar between FGA and SGA: haloperidol, olanzapine, and quetiapine significantly reduced Arc/Arg3.1 gene expression compared to controls following a single injection [54]. Since D_2_R is not highly expressed in the frontal cortex, the possibility exists that these drugs act on cortical D_2_R extremely sensitive to D_2_R antagonism [67]. Another possibility may derive from the notion that both olanzapine and quetiapine increase cortical dopamine release, at variance from haloperidol; accordingly, it could be speculated that such released dopamine would selectively, or primarily, interact with cortical D_2_R reducing Arc/Arg3.1 mRNA levels, as observed with the D_2_R agonist quinpirole.

The acute treatment with the SGA amisulpride, which can be almost considered a pure D_2_R/D_3_R antagonist, also acting at presynaptic level [68], revealed a more attenuated profile of striatal induction when compared to haloperidol [69], closer to the striatal profile induced by olanzapine. The acute treatment with SGA lurasidone, which possesses a heterogeneous receptor profile in which a powerful antagonism of the 5HT_7_ receptor stands out above all, revealed a regionally distinct activation pattern. In fact, at the doses of 1 and 3 mg/kg, Arc/Arg3.1 mRNA levels were increased in the hippocampus, unchanged in the PFC, while increased in the striatum at the higher dose of 10 mg/kg [70]. While the effect in the striatum implies the predominant antagonism on D_2_R extensively recruited at the highest dose, the hippocampal effect indicates, at least in part, a dose-dependent effect on Arc/Arg3.1 mRNA levels, which may be extremely relevant after a single treatment. Since there was no effect in the PFC, it is difficult to hypothesize that such an increase could be ascribed to the interaction with a specific receptor subtype but, rather, to the increased synaptic levels of neurotransmitters, for instance, glutamate, known to modulate Arc/Arg3.1 mRNA levels. Another explanation for the effect of the lower dose of lurasidone suggests a role of 5HT_7_ receptors in Arc/Arg3.1 modulation, which is still unexplored. Instead, the striatal up-regulation observed is likely to be related to the antagonism of D_2_R, as previously discussed.

Asenapine, a second-generation drug characterized by a broad receptor occupancy with an equal potency of D_1_R and D_2_R blockage [71], displayed a dose-dependent profile of Arc/Arg3.1 mRNA modulation; in fact, incremental doses of asenapine increased Arc/Arg3.1 mRNA levels in the striatum and nucleus accumbens (NAc), whereas at the cortical level, the lowest and the highest doses of the drug (0.05 and 0.3, respectively) did not alter Arc/Arg3.1 gene expression, while the intermediate dose (0.1 mg/kg) increased Arc/Arg3.1 mRNA levels [72]. This effect may be due to the elevation of dopamine release in the cortex and basal ganglia observed at the 0.1 mg/kg dose, with higher doses presumably reaching a plateau in dopamine efflux [73]. It is interesting to point out that sertindole, an AP with relatively high affinity at D_2_R *in vitro* not associated with high D_2_R occupancy *in vivo* and virtually devoid of EPS liability in humans [74], does not alter Arc/Arg3.1 mRNA levels [75]. Interestingly, a drug still under clinical development, SEP-363856, mainly acting at the presynaptic serotonergic receptor 5HT_1_ and trace amine-associated (TAAR1) receptors [76], increased Arc/Arg3.1 mRNA levels in the PFC at the different doses used while increasing selectively in the ventral hippocampus (vHip) at the lowest dose with no effects at any dosage in the dorsal hippocampus (dHip) and striatum [77]. Given the specific receptor profile of SEP-363856, the possibility exists that Arc/Arg3.1 up-regulation may be due to the activation of 5HT_1A_ receptors since the selective serotonin 5-HT_1A_ receptor agonist ((+)-8-OH-DPAT) enhances Arc/Arg3.1 mRNA levels [78]; however, we cannot rule out the possibility that this effect may be due, at least partially, to the stimulation of TAAR_1_ receptors.

Indeed, different APs induce distinct temporal and regional molecular profiles of Arc/Arg3.1 expression within the brain, suggesting that diverse grades of D_1_R and D_2_R subtype activation/blockade may affect Arc/Arg3.1 expression differently. The several lines of evidence discussed above suggest that the duration of Arc/Arg3.1 elevation following acute AP injection may help distinguish between the different classes of AP and represent a reliable index of low/high propensity to cause extrapyramidal side effects, a feature relevant for drug screening.

### 3.2 Repeated Treatments

A different situation can be depicted following repeated exposure to APs. Chronic treatment with haloperidol revealed a pattern opposite to acute treatment in the striatum (reduction *vs*. enhancement) [54] at 2 and 24 hours after the last injection, an effect shared by olanzapine. Notably, in the frontal cortex, haloperidol did not cause any alteration at both time points following repeated exposure, in line with Collins and associates who performed immunohistochemistry [79]. Differentially, olanzapine still reduced Arc/Arg3.1 mRNA levels 2 hours after the last injection, similarly to the single treatment, presumably an effect of the last injection [54], as such effect vanished 24 hours later. Moreover, at doses of haloperidol lower than 1 mg/kg (*i.e*., 0.25, 0.5, 0.8 mg/kg), no effects were observed in the striatum, but a significant reduction in the frontal cortex, suggesting again that the dosage of APs, indicative of the occupancy of presynaptic *vs.* postsynaptic dopamine receptors, does indeed play a role on Arc/Arg3.1 mRNA levels [80]. Of course, other mechanisms may come into play following repeated exposure to haloperidol or olanzapine, as glutamate neurotransmission is differently altered following prolonged administration of these drugs [81]. Additionally, serotonergic mechanisms might also partially explain the reduced cortical Arc/Arg3.1 mRNA levels through the blockade of the 5-HT_2A_ receptors [82].

Interestingly, prolonged treatment with quetiapine reduced Arc/Arg3.1 mRNA levels when rats were sacrificed 2 hours after the last treatment, similarly to the single treatment in the frontal cortex, again suggesting the effect of the last injection, but not in the striatum [54], presumably because quetiapine has a lower D_2_R affinity than olanzapine [83]. In addition, Collins and associates found a reduction of Arc/Arg3.1 mRNA levels in the cingulate cortex of rats chronically exposed to the SGA clozapine [79]. Such a different Arc/Arg3.1 mRNA level modulation profile indicates that neuroadaptive mechanisms occur following repeated AP exposure. It must be considered that Arc/Arg3.1 strictly interacts with type II calcium calmodulin kinase (CaMKII) [84, 85], which is reduced following repeated exposure to haloperidol or olanzapine [86]: this indicates that change in Arc/Arg3.1 expression may represent a piece of the puzzle contributing to the striatal neuroplastic modifications induced by prolonged AP exposure.

Recently, a novel generation of APs has been developed, among which we count aripiprazole, brexpiprazole, and cariprazine. They are D_2_R partial agonists with different receptor affinities besides being antagonists of the 5HT_2A_ serotonergic receptors. The partial agonist feature at D_2_R of aripiprazole, the prototype of this drug category, implies that, under situations of mesolimbic hyperdopaminergia, aripiprazole would set up an antagonistic action whereas, under conditions of low cortical dopamine, the drug would stimulate D_2_R. We have shown that repeated exposure to aripiprazole has increased Arc/Arg3.1 expression in the striatum, PFC (the highest peak) and hippocampus [87] suggesting a different pattern of induction with respect to repeated treatments with olanzapine or quetiapine that rescued the expression of Arc/Arg3.1 [54]: such difference may be due to the partial agonist activity on D_2_R that may avoid the increased expression of D_2_R following repeated administration. Interestingly, when the authors exposed rats with a history of aripiprazole treatment to acute stress, they observed that stress was not able to further increase Arc/Arg3.1 mRNA levels, as instead shown in naïve animals, pointing to the ability of aripiprazole to buffer cell responsiveness in an anatomical specific manner under a challenge. These data show the functional selectivity action of aripiprazole at D_2_R and allow us to speculate that the Arc/Arg3.1 mRNA levels may represent a marker of the dynamic modulation of dopaminergic neurotransmission exerted by aripiprazole as a function of the dopaminergic tone. However, we cannot rule out the possibility that, since aripiprazole has a broad multi-receptor profile drug [88], other receptors could contribute to such an effect.

The repeated treatment with asenapine revealed the critical role of brain structural changes occurring at the post-synaptic density [80], an electron-dense zone where post-synaptic glutamatergic action occurs and where interactions between glutamate and dopamine neurotransmission are established [89]. The most interesting result was indeed the strong reduction caused by asenapine on cortical Arc/Arg3.1 mRNA levels that could be ascribed to its strong action as a 5-HT_2A_ receptor antagonist. In fact, asenapine reduced Arc/Arg3.1 mRNA level more than olanzapine, a less potent 5-HT_2A_ receptor antagonist [90, 91]. Notably, in the striatum, where D_2_R are densely expressed, haloperidol increased Arc/Arg3.1 mRNA level at a greater extent than asenapine and olanzapine [80] due to the potent blockage of D_2_R.

The modulation of Arc/Arg3.1 mRNA levels was also investigated following repeated exposure to lurasidone. Evidence exists showing that Arc/Arg3.1 mRNA levels were increased in all the brain areas investigated, with changes that strongly depended on the doses employed [70]. Interestingly, some of these effects were different from the acute effects, suggesting different sustaining mechanisms. In the hippocampus, Luoni and colleagues (2014) found a different effect [70] when compared to other SGA [54], a difference that might depend upon the strictly inherent receptor profile of each drug. In the striatum, these authors found a peculiar pattern of Arc/Arg3.1 modulation, as only the lowest dose of lurasidone (1 mg/kg) increased the expression of the effector early gene, suggesting a mechanism other than blockade of D_2_R. One possibility may derive from the specific blockade of 5HT_7_ receptors, which has been shown to augment serotonin release [92], an effect that may increase Arc/Arg3.1 mRNA levels [93]. It is important to note that lurasidone also shows AD properties, and some effects observed at the lowest doses could be related to such activity. Accordingly, the serotonin and noradrenaline reuptake inhibitor duloxetine shows a similar increase in the PFC [94]. The effects of the dose of 10 mg/kg in different experiments have also been shown by our group [95].

Recent evidence has been published on the novel drug blonanserin and its effects on Arc/Arg3.1 mRNA levels. Blonanserin is an SGA drug acting as an antagonist of D_2_R, D_3_R, and 5-HT_2A_ serotonin receptors [96]. Exposure to blonanserin at 0.3 mg/kg induced Arc/Arg3.1 mRNA levels in both dHip and vHip with no effects in the medial PFC (mPFC) [97], in line with previous data [87, 98] showing a significant increase in Arc/Arg3.1 mRNA levels in the dHip. Notably, when rats were exposed to chronic mild stress, Arc/Arg3.1 mRNA levels were significantly reduced in the mPFC and vHip, an effect that was otherwise reverted by the 3 mg/kg dose of blonanserin [97], supporting the notion that Arc/Arg3.1 is one of the synaptic markers normalized by APs under stressful conditions.

Thus, while acute exposure to AP drugs relies on rapid effects primarily due to the interaction with specific receptor subtypes, the effect of a long-term administration of AP drugs is not the mathematical sum of multiple, single treatments. This is demonstrated by the modulation of Arc/Arg3.1 expression since prolonged exposure to APs often causes different, sometimes opposite, effects compared to a single administration. The diversity of the chronic from the acute effects may also depend upon the different brain areas analyzed: for example, the exposure to a single dose of an FGA drug likely causes a marked effect on the expression of Arc/Arg3.1 mRNA levels, especially in areas with a higher density of dopaminergic receptors such as the striatum whereas the repeated exposure of the same drug may cause changes also in other brain areas such as the hippocampus, a region with scarce presence of these receptors. Furthermore, the dosage of AP drugs is also a reason why different effects may be observed between acute and repeated treatments, mostly because at a low dosage, the drug may interact with receptors other than those with which it interacts acutely, or it can interact with receptors located presynaptically, instead of postsynaptically, thus generating different effects at the molecular level.

We also need to consider that most of the experimental studies examined have involved treatments with APs on wild-type animals and not animal models of psychosis. However, some experiments have included treatments that mimic conditions resembling, at least partially, some symptoms of schizophrenia. To this end, Nakahara and colleagues (2000) have measured the expression of Arc/Arg3.1 mRNA levels in rats previously exposed to phencyclidine (PCP), an antagonist of glutamate NMDA receptors used to mimic positive, negative, and cognitive symptoms of schizophrenia [99]. These authors have shown that pretreatment with clozapine, olanzapine, or risperidone prevented PCP-induced Arc/Arg3.1 mRNA levels in the PFC, an effect not shared by haloperidol [99]. Conversely, previous treatment with haloperidol increased the striatal Arc/Arg3.1 mRNA levels. These data demonstrate that FGAs may be easily distinguished from SGA when using the appropriate animal model; this notion confirms previous evidence from our group showing that olanzapine, but not haloperidol, under reduced NMDA activity, a feature of schizophrenia, was able to up-regulate the expression of the neurotrophin BDNF, a protein closely related to Arc/Arg3.1 [100], overall indicating distinct neuroplastic actions of FGA and SGA under specific experimental conditions. However, we also have to consider the timing of the exposure of rats to PCP: if rats are exposed to PCP when adolescent, then PCP reduces Arc/Arg3.1 mRNA levels in the PFC, instead of increasing it as in adult rodents, in line with the theory of hypo-frontality of schizophrenia and, maybe, the effect of AP treatment could be different [52].

## 4 EFFECT OF ANTIDEPRESSANT DRUGS ON ARC/ARG3.1 MODULATION

At variance from schizophrenia, genetic alterations of Arc/Arg3.1 have not been linked to major depression. However, evidence exists showing alterations of Arc/Arg3.1 expression in experimental models of this disorder [100-102]. Differently from APs, whose single exposure may be critical in the attenuation of the acute symptoms of the disorder, acute exposure to ADs does not hold clinical significance as these drugs need at least 8 weeks before eliciting AD activities, which is indicative of neuroadaptations over the course of repeated drug exposures. Accordingly, the acute modulation of proteins is not critical for the action of ADs, and, in general, it is used just to show that the effect of a given chronic treatment is not due to the effect produced by the last drug exposure before sacrificing the rodent. Furthermore, such neuroplastic mechanisms also involve the modulation of Arc/Arg3.1, as repeated AD exposure up-regulates Arc/Arg3.1 expression in selected brain regions of the rat forebrain, which might play a role in long-term changes in synaptic function [93].

The most widely used AD is indeed fluoxetine, which selectively inhibits the serotonin transporter, thus increasing serotonin levels in the synaptic cleft. De Foubert and coworkers (2004) have shown that only 14 days of repeated treatment with fluoxetine increased the expression of Arc/Arg3.1 mRNA levels, with no effects after 4 or 7 consecutive days of administration [103, 104], suggesting a role for Arc/Arg3.1 in the therapeutic activity of fluoxetine. In line, chronic, but not single, administration of fluoxetine increased Arc/Arg3.1 mRNA levels in the hippocampus, with a not significant trend toward an increase in the PFC [105]. Notably, such a pattern of expression is similar to that displayed in both manuscripts by the neurotrophin BDNF, a widely accepted signature for most ADs. These results suggest that Arc/Arg3.1 upregulation following 14 days of fluoxetine treatment may be secondary to the increase of BDNF expression, which stimulates the pathway for Arc/Arg3.1 expression.

Eriksson and colleagues (2012) exposed a genetic rat model of depression, *i.e*., Flinders sensitive line (FSL) rats, to different AD drugs such as escitalopram, a selective serotonin reuptake inhibitor, and nortriptyline, a tricyclic AD that blocks serotonin and norepinephrine transporters [102]. FSL rats displayed reduced Arc/Arg3.1 mRNA levels in the PFC and hippocampus at baseline, an effect that caused emotional memory deficit. Notably, repeated administration of escitalopram, but not nortriptyline, improved memory in these rats, an effect presumably linked to restored Arc/Arg3.1 mRNA levels. These results suggest that Arc/Arg3.1 mRNA levels are more sensitive to the synaptic levels of serotonin and not norepinephrine, an effect presumably involving the stimulation of 5-HT_4_ receptors.

Vortioxetine is a novel multimodal AD that combines different features as it is a 5-HT_1A_ receptor agonist, a 5-HT_1B_ receptor partial agonist, an antagonist for 5-HT_3_, 5-HT_7_, 5-HT_1D_ receptors, and a serotonin transporter inhibitor [106]. We have shown that repeated exposure to vortioxetine tended toward an increase of Arc/Arg3.1 mRNA levels in the vHip [107], a very specific anatomical selectivity that could be due to 5-HT_1A_ receptors, highly expressed in both CA1 and CA3 subregions, [108, 109] and, presumably, also in part to the increased extracellular levels of serotonin occurring in vHip [110]. However, the vortioxetine-induced increase in the vHip modulated the response to stress by preventing Arc/Arg3.1 mRNA level elevation, thus influencing the rapid neuronal response to a challenging experience. These data may be functionally relevant as the vHip modulates emotional processes, which are compromised in depressed patients [111]. Chronic treatment with vortioxetine in young and middle-aged rodents [112] increased Arc/Arg3.1 mRNA and protein levels in the cortical synaptosomes from both groups. This may result from increased glutamate neurotransmission since vortioxetine activates its release [113] or serotonin neurotransmission [114]. As in cell culture preparations, it is possible to disentangle potential mechanisms of drug action; these authors point to the 5-HT_3_ antagonism-mediated attenuation of GABAergic inhibitory control of glutamate signaling exerted by vortioxetine as the mechanism responsible for the elevation of Arc/Arg3.1 synthesis and, likely, of the increased performance in vortioxetine-treated rats [115, 116]. We have shown that repeated duloxetine exposure modulates the Arc/Arg3.1 expression in selected rat brain regions [94]. Interestingly, the effects of duloxetine vary depending on the time of sacrifice after the last injection (1 hour and 24 hours); for instance, in the frontal cortex, Arc/Arg3.1 mRNA levels are markedly induced when rats are sacrificed 1 hour after the last treatment while back to control levels 24 hours later; notably, a single administration of duloxetine caused a significant reduction of Arc/Arg3.1 mRNA levels in the frontal cortex suggesting that the marked increase observed 1 hour after the last administration cannot be ascribed to the last injection, but rather to the adaptive mechanisms set in motion by the repeated treatment regimen. Furthermore, by exposing rats chronically treated with duloxetine to acute stress, we did not find any change at the transcriptional level, while duloxetine pre-treatment counteracted the stress-induced rapid synaptic reduction of Arc/Arg3.1 protein levels in the frontal cortex. The evidence that both vortioxetine and duloxetine prevented the stress-induced modulation of Arc/Arg3.1, despite in different brain areas, further points to the effector early gene as critical in the remodeling processes following repeated AD exposure.

Focusing on venlafaxine, a mixed serotonin-norepin-ephrine reuptake inhibitor that binds and blocks both the serotonin and noradrenaline transporters [117], Serres and colleagues (2012) demonstrated that chronic administration of this drug enhanced Arc/Arg3.1 mRNA levels in the cingulate and parietal cortex when rodents were sacrificed 2 hours after the last injection, whereas such increase persisted only in the parietal cortex when measured 16 hours later [118]. Notably, no effects were observed in the hippocampus of venlafaxine-treated rats. This result is extremely interesting given the evidence that venlafaxine activated cortical areas during cognitive tests in depressed patients [119]; in addition, repeated stress causes neuronal atrophy in the cingulate cortex, which might be reversed by venlafaxine through modulation of Arc/Arg3.1 expression [120]. Moreover, in a challenging situation, we recently found that chronic venlafaxine treatment enhanced Arc/Arg3.1 gene expression following acute restraint stress in both dHip and vHip, an effect not observed in control rats exposed to the same stress [121].

A novel AD that recently came to the market is agomelatine. Agomelatine has a peculiar receptor profile as it is a melatonin MT_1_/MT_2_ receptor agonist and 5-HT_2C_ receptor antagonist. We compared the effects of long-term treatment with agomelatine to another AD venlafaxine. Interestingly, both drugs increased Arc/Arg3.1 mRNA levels in the hippocampus while reducing it in the PFC. Notably, such changes were observed when rats were sacrificed 1 hour after the last injection but waned 16 hours later. The similar profile of Arc/Arg3.1 modulation following repeated exposure to AD drugs with different mechanisms suggests that the modulation of Arc/Arg3.1 mRNA levels is not strictly correlated to the unique receptor profile of these drugs but, rather, relies more on neuroadaptive changes taking place following prolonged AD treatment [122]. Notably, in a more dynamic situation, Boulle and associates (2014) showed that agomelatine restored stress-induced increase of Arc/Arg3.1 mRNA levels to the levels of non-stressed rodents [123] suggesting that agomelatine may normalize neuronal activity.

## 5 GENERAL CONSIDERATIONS FOR ANTIPSYCHOTIC AND ANTIDEPRESSANT EFFECT ON ARC/ARG3.1

In an attempt to draw some conclusions regarding the effect of AP and AD drugs on Arc/Arg3.1 modulation, it is important to underline that, as an IEG, Arc/Arg3.1 is uniquely positioned to reconcile genetic variations and environmental influences on psychiatric disorder susceptibility. Thus, the possibility exists that genetic variations may reduce the activation of Arc/Arg3.1 following environmental stimuli, hence lowering the response of the system under such a dual combination. This may result in widespread changes involving, for instance, spine density reductions as well as impaired memory and cognitive response, commonly observed in schizophrenic and depressed patients, thus increasing the risk of developing these disorders. Following this line of reasoning, dysfunctions of Arc/Arg3.1 may impair the normal neurobiological response to stress, paving the way for the manifestation of schizophrenic or depressive symptoms. In other words, genetic variations of Arc/Arg3.1 may predict the response to environmental challenges. Thus, it has been hypothesized the presence of an activity-dependent biological pathway, which includes depression and schizophrenia candidate genes, and that Arc/Arg3.1 is part of it [124]: the possibility exists that AP or AD drugs may oppose the derailment of this biological pathway through their action on Arc/Arg3.1 and on other elements of this pathway of neuroplasticity.

The mechanisms coupling drug administration to Arc/Arg3.1 transcription are not easy to elucidate. In fact, there are no data available explaining that the effects of AP or AD drugs on Arc/Arg3.1 are due to selective actions on its synthesis, turnover, degradation and/or to the combinations of some of these potential mechanisms. In addition, AP or AD drugs may influence the trafficking of Arc/Arg3.1 into the dendrite of an activated synapse, thus altering its subsequent translation. Another option is that drug-induced alterations in Arc/Arg3.1 transcription might be due to rapid changes in synaptic properties coupled to *de novo* translation involving, for instance, ribosome translocation [125]. AP or AD drugs might also impact neurogenesis, which is critical for memory, a task regulated by Arc/Arg3.1.Accordingly, it has been shown that acute or chronic stress reduces granule cell precursor proliferation [125, 126], an effect reversed by antidepressant treatment [127, 128].

Indeed, it has been demonstrated that Arc/Arg3.1 expression is tightly regulated by the BDNF-TrkB signaling pathway. While most of the ADs available rely on mechanisms interfering with the reuptake of monoamines, recent evidence shows that they act by directly binding to the high-affinity receptor of BDNF, *i.e*., TrkB [129]. This suggests that enhancing BDNF release is, indeed, a common mechanism for the action of ADs, and considering the direct relationship between BDNF and Arc/Arg3.1 [27], it can be hypothesized that psychotropic drugs promoting BDNF release positively influence Arc/Arg3.1 expression.

The most used antidepressant is fluoxetine, which increases serotonin levels in the synaptic cleft and up-regulates Arc/Arg3.1 expression, as discussed above. However, serotonin may affect Arc/Arg3.1 expression also indirectly; in fact, since it is known that serotonin influences the responses to glutamate [130], it may be hypothesized that drugs enhancing serotonergic neurotransmission may increase Arc/Arg3.1 expression through indirect potentiation of glutamatergic functions. One of the mechanisms through which glutamate, *via* NMDA transmission, may increase the expression of Arc/Arg3.1 relies on increased phosphorylation of the transcription factor CREB [131], which is known to be activated by AP or AD drugs [132]. The only exception to the delayed effect brought about by AD drugs is represented by ketamine, a non-competitive antagonist of glutamate NMDA receptors, which shows a rapid onset and sustained antidepressant effect. Of note, we have recently demonstrated that a single infusion of a translational dose of ketamine activates the BDNF-dependent eukaryotic elongation factor 2 kinase [133], which is a requirement for increased Arc/Arg3.1 translation [134], providing an additional mechanism to sustain Arc/Arg3.1 expression. Interestingly, serotonin may also interact with other receptors to enhance Arc/Arg3.1 expression; for instance, agomelatine, an antidepressant exhibiting antagonistic properties at 5-HT_2C_ receptors together with agonistic features toward melatonin MT_1_ and MT_2_ receptors, when administered acutely [135] or chronically [122] elevates Arc/Arg3.1 mRNA levels.

## 6 EFFECT OF PSYCHOSTIMULANTS ON ARC/ARG3.1 MODULATION

When analyzing the effects of psychostimulants on Arc/Arg3.1 expression/modulation, we must distinguish between acute and repeated exposure since the effects of psychostimulant administration on Arc/Arg3.1 depend on the duration of the treatment itself. In fact, a single psychostimulant exposure may rapidly alter neuronal activity as a consequence of rapid blockade of neurotransmitter transporters and, therefore, activate Arc/Arg3.1, an effect that is immediately reflected in terms of the amount of neurotransmitter availability in the synaptic cleft, thus boosting postsynaptic receptor activation. The repeated exposure, instead, relies on long-term changes in functional and structural plasticity. Furthermore, the repeated treatment, when abruptly interrupted, determines the phenomenon of the so-called withdrawal, which can determine functional and structural changes that, in turn, may include the modulation of Arc/Arg3.1.

About the single exposure to cocaine, Arc/Arg3.1 can be differently modulated depending on the timing of exposure: the effect of psychostimulants, and consequently, their effects on Arc/Arg3.1 may change if they are administered during adolescence, *i.e*., when brain development is actively ongoing, or at adulthood when brain development is completed. We have demonstrated that a single exposure to cocaine during adolescence reduces cortical Arc/Arg3.1 protein levels, whereas no effects were observed when it was administered to adult rats [136], further suggesting the higher sensitivity of the adolescent brain to even a single cocaine injection. The rapid modulation of Arc/Arg3.1 protein is indeed intriguing since it also interacts with the actin cytoskeleton, which is altered by adolescent cocaine exposure, presumably explaining the spine remodeling induced by the single administration [137]. Interestingly, Salery and colleagues (2017) revealed that a single cocaine injection is sufficient to rapidly induce and accumulate Arc/Arg3.1 in the nucleus of striatal neurons in mice [138]. These authors provided evidence that the accumulated Arc/Arg3.1 acts as a brake on gene regulation, thus influencing chromatin remodeling and inducing molecular and behavioral responses to cocaine by inhibiting activity-dependent transcription of genes.

Repeated exposure to cocaine during adolescence, *i.e*., between postnatal day 28 to 42, leads to a reduction of Arc/Arg3.1 mRNA and protein levels in both homogenate and synaptic fraction of infralimbic (IL) and prelimbic (PL) cortices, indicating a baseline reduction of neuronal activity in the overall mPFC [139]. Exposure of saline- and cocaine-treated adolescent rats to the novel object recognition (NOR) test, a cognitive task, increased Arc/Arg3.1 mRNA levels in the PL cortex in both groups, indicating an activation of this cortical subregion induced by the test. In the IL cortex, cocaine exposure led to the activation of Arc/Arg3.1 levels following NOR exposure, an effect not observed in saline-treated rats. These results suggest that, under normal conditions, the IL cortex is not recruited to perform the test, whereas it is abnormally engaged in rats with a history of developmental cocaine exposure [139]. This evidence also suggests that adolescent cocaine exposure has selectively sensitized the response of the IL cortex to a novel environmental situation. These data are in line with another manuscript from our laboratory showing that repeated exposure to cocaine during development reduced Arc/Arg3.1 mRNA levels in the mPFC, but in response to acute swim stress, Arc/Arg3.1 mRNA levels are significantly upregulated [140]. Thus, it is possible to speculate that reduced baseline Arc/Arg3.1 mRNA levels in the mPFC of cocaine-exposed rats may represent a marker of hypersensitivity to challenging events, whether cognitive or stressful in nature. In addition, given its nature of being quickly activated, a deficit in Arc/Arg3.1 expression may alter the formation of new memories, thus reducing the adaptability to an external stimulus. These considerations widen the importance of dissecting the subregional dependent Arc/Arg3.1 modulation in experimental animals.

Several lines of evidence have highlighted that stress and cocaine are often partners in crime and share several targets. One of the early targets of such a duo is indeed represented by the modulation of IEG expression [141-143]. We found that stress interacts with cocaine to promote changes in Arc/Arg3.1 mRNA levels in the mPFC, further confirming that this brain region is extremely sensitive to the effects of stress, as previously observed [144]. In this manuscript, we found that stress influenced Arc/Arg3.1 expression following single cocaine exposure in a different way depending on whether the stress was acute or repeated [145]. In fact, acute stress fueled the increase in Arc/Arg3.1 mRNA levels caused by cocaine, whereas chronic stress blunted such a response. Notably, repeated stress reduced baseline Arc/Arg3.1 mRNA levels similarly to what above mentioned following chronic cocaine exposure, suggesting that exposure to a long-term event, be it repeated cocaine exposure or repeated stress, alters Arc/Arg3.1 expression; accordingly, the reduction in the expression of this effector gene can be considered a marker of long-term exposure to adverse events in mPFC.

Another variable to be taken under consideration when examining the modulation of Arc/Arg3.1 mRNA levels relies on the environmental context in which the psychostimulant is administered [146, 147]. Klebaur and associates (2002) administered either saline or amphetamine in the rat’s home cage or in a distinct test environment and found that in some brain regions such as PFC, caudate-putamen and core of the NAc, Arc/Arg3.1 mRNA levels were increased under both experimental conditions, but the magnitude of the effect was more pronounced when amphetamine was administered in a distinct environment. Conversely, in the shell of the NAc, Arc/Arg3.1 mRNA levels were upregulated only when amphetamine was given in a distinct environment [148, 149]. Given the neuroplastic and structural role of Arc/Arg3.1, the evidence that Arc/Arg3.1 mRNA levels are modulated in a way that heavily relies on contextual stimuli may represent a mechanism set in motion by the environment to modulate multiple forms of experience-dependent synaptic plasticity.

The rapid modulation of Arc/Arg3.1 mRNA levels can also be instrumental in understanding whether Arc/Arg3.1 could be involved in drug-seeking. An elegant manuscript from Penrod and colleagues (2020) has recently shown that Arc/Arg3.1 influences cocaine self-administration in mice, suggesting that this effector gene plays a role as a regulator of drug-taking susceptibility [111]. Accordingly, we employed a contingent paradigm of cocaine treatment, the so-called yoked control-operant paradigm, *i.e*., a modality of cocaine administration that allows the separation of the direct pharmacological effects of cocaine from those associated with active drug self-administration. Under these experimental conditions, we observed that Arc/Arg3.1 mRNA levels are selectively upregulated in the mPFC of rats actively self-administering the psychostimulant [143], with no effects on classical IEGs such as *Zif268*. These results associate Arc/Arg3.1 mRNA elevation in mPFC with motivation driven by voluntary self-administration, a sort of Arc/Arg3.1-mediated conditioned plasticity following contingent cocaine administration. Although we cannot rule out the possibility that such selective increase could be due, at least in part, to increased cortical neuronal activity, it may be associated with expectation consequent to the exposure to a goal-oriented behavior paradigm [150] or, alternatively, to the instrumental training *per sé*. Thus, it appears that the elevation of Arc/Arg3.1 mRNA levels may be related to learning-dependent mechanisms, which occur rapidly after the first presentation of the drug. The role of motivation in triggering elevations of Arc/Arg3.1 mRNA levels is further demonstrated by experiments from Zavala and associates (2008), who trained rats to self-administer cocaine followed by extinction training, during which cocaine-seeking behavior was progressively reduced [151]. They found that exposure to cues that had been previously paired with cocaine infusions during training raised Arc/Arg3.1 mRNA levels primarily at the cortical level, pointing to Arc/Arg3.1 as critical for conditioned plasticity associated with incentive motivational effects of cocaine cues. Notably, this mechanism has also been observed following exposure to contextual cues associated with nicotine [152] or food [153], suggesting Arc/Arg3.1 as a common denominator of processes characterized by motivational stimuli. In addition, we cannot rule out the possibility that Arc/Arg3.1 mRNA level elevation might be due to cocaine withdrawal or extinction training, as we have demonstrated following long-term withdrawal [154].

It is interesting to point out that Salery and colleagues (2017) reported that Arc/Arg3.1 knockout mice showed cocaine-conditioned place preference [138] suggesting that its removal does not alter the rewarding properties of cocaine; however, it has to be noted that such preference was manifested at doses that are normally not effective in wild-type counterparts, as shown by Contarino and associates (2017) [155], raising the possibility that Arc/Arg3.1 removal might enhance the rewarding effects of cocaine.

Arc/Arg3.1 mRNA may also be considered a marker of the memory reconsolidation process driven by psychostimulant administration. In fact, Shi and coworkers (2022) have recently shown that Arc/Arg3.1 mRNA levels are enhanced 60-120 min after retrieval of cocaine [156], in line with previous work involving the analysis of Arc/Arg3.1 protein level [157], suggesting that the reactivation of cocaine contextual memory may engage Arc/Arg3.1 among the other mechanisms.

Cocaine regulates the expression of Arc/Arg3.1 following repeated exposure in a way that may also depend on the short- or long-term withdrawal before sacrifice. We have shown that rats exposed to cocaine during adolescence and sacrificed at postnatal day 90, *i.e*., after 48 days of withdrawal, exhibited increased levels of Arc/Arg3.1 in the whole homogenate and the nuclear fraction of the mPFC, suggesting that the nuclear upregulation may contribute to the overall Arc/Arg3.1 enhancement [154]. We hypothesized that such increase could be mediated by reduction of fragile X mental retardation gene (FMR1) expression, which normally inhibits Arc/Arg3.1 translation, coupled with reduced expression of Ubiquitin-protein ligase E3A (Ube3a) that usually causes Arc/Arg3.1 protein degradation *via* ubiquitination [158] and increased GRM5 expression, which physiologically promotes Arc/Arg3.1 translation and synthesis [134]. This evidence highlights that the machinery regulating Arc/Arg3.1 expression is finely tuned through the involvement of independent mechanisms, leading to enduring changes that become manifest long after the end of cocaine treatment. Of note, such regulation of Arc/Arg3.1 expression overlaps with that observed for the neurotrophin BDNF that we found increased after 48 days of abstinence [159]: this is not unexpected since both molecules mediate the adaptive changes set in motion by psychostimulants [160], allowing to hypothesize that the binomial BDNF-Arc/Arg3.1 may contribute to the incubation of cocaine craving following a long period of abstinence [136].

Arc/Arg3.1 mRNA levels appear to vary also when psychostimulants other than cocaine are used, such as cathinones [161]. In 2017, we employed two different cathinones, *i.e*., 3,4-methylenedioxypyrovalerone (MDPV) and 1-phenyl-2-(pyrrolidin-1-yl) pentan-1-one (α-PVP), often available in the clandestine market as bath salts or fertilizers. These cathinones cause changes in Arc/Arg3.1 mRNA levels that depend on the brain area investigated. In fact, in the mouse striatum, MDPV induced an increase of the effector gene more persistent than α-PVP, whose effect wanes more rapidly [162], presumably due to the highest density of dopamine transporter present in the striatum [163] to which MDPV has higher affinity. In the hippocampus, instead, α-PVP appears to be unable to trigger Arc/Arg3.1 mRNA elevation, at variance from MDPV, which leads to a marked increase that, however, is not immediate, raising the possibility that it may play a role in secondary drug-related neuroadaptive mechanisms, perhaps glutamatergic in nature [137].

Also, amphetamine or methylphenidate modulates Arc/Arg3.1 mRNA levels. Both these drugs have been used for Attention Deficit Hyperactivity Disorder (ADHD). Biever and associates (2017) found that repeated exposure to amphetamine elevates Arc/Arg3.1 mRNA levels in the striatum and NAc, an effect primarily mediated by D_1_-positive medium spiny neurons and NMDA-expressing neurons in striatal regions [164] suggesting that Arc/Arg3.1 integrates dopamine D_1_- and glutamate NMDA-mediated pathways following amphetamine. In line with these observations, we found that amphetamine exposure during adolescence enhanced Arc/Arg3.1 expression, which lasted at least one month after the end of treatment [160], suggesting that early in-life exposure may lead to enduring changes in Arc/Arg3.1 expression. Banerjee and coworkers (2009) interestingly found that the modulation of Arc/Arg3.1 mRNA levels differed depending on the period of psychostimulant exposure, whether during juvenile age or adulthood, in a brain region-dependent fashion: the authors found a decrease in the brain of juveniles and an increase in adults [165]. Gronier *et al*. (2010) confirmed the age-dependent effects of methylphenidate on Arc/Arg3.1 in the PFC, reflecting developmental differences in brain responsiveness presumably because of the immature state of neurotransmitters regulating Arc/Arg3.1 expression in the PFC of juvenile rats [166]. Similar data were also collected by Chase and colleagues (2007) [167]. These data are of some interest considering that methylphenidate is widely used in children with ADHD and may suggest that, at least in part, the long-term detrimental effects of this amphetamine-like compound may be related to Arc/Arg3.1 down-regulation.

## CONCLUSION

Recent manuscripts have added novel, critical information about the structure, function, and mechanisms of induction of Arc/Arg3.1.However, the information about its role in the modulation of brain functions is still fragmented. Arc/Arg3.1 is indeed a fascinating, enigmatic, and versatile protein with a unique role in bringing together drug-induced neuronal activity and neuroplasticity. Despite there is no evidence causally linking Arc/Arg3.1 to psychiatric disorders, based on the existing literature, it is possible to hypothesize that alterations of Arc/Arg3.1, together with alterations of other markers of synaptic plasticity, could be part of the intricate complex of structural and functional changes that contribute to the pathogenesis of these disorders. This seems to be true also for psychiatric disorders not covered in this review as recent evidence shows Arc/Arg3.1 alterations also in animal models of anorexia nervosa [168], expanding the contribution of this effector gene also to the regulation of homeostatic and hedonic features of feeding behavior.

A common ground of these disorders is indeed represented by altered cognition, which can persist even after significant improvement of the main symptoms. Accordingly, cognitive deficits represent an endophenotype of these disorders or even a predictive feature of the functional outcome or treatment response [138, 139]. The analysis of Arc/Arg3.1 modulation may give different information depending on the duration of the specific manipulation: in fact, the rapid modulation of Arc/Arg3.1 mRNA levels may inform about the cellular responsiveness ongoing in specific brain regions whereas long-term modulation of Arc/Arg3.1 protein levels may reflect either the extent of brain remodeling or its capacity to modulate functions such as LTP, LTD and synaptic scaling, each fundamental for proper cognitive functions. Given the critical role of Arc/Arg3.1 in cognition, the possibility, thus, to modulate Arc/Arg3.1 expression and ameliorate such disorder-related impairments in cognitive abilities further points to the importance of the still unexplored critical role of Arc/Arg3.1 in the brain [169, 170].

A further important concept to discuss relies on the notion that elevation of Arc/Arg3.1 expression may be indicative of mechanisms of coping behavior. For instance, exposure to a novel environment activates a series of changes involving up-regulation of Arc/Arg3.1 as a critical signature in the mPFC [171-174]. In addition, acute self-administration of cocaine up-regulated Arc/Arg3.1 expression in the mPFC [124]. The intrinsic peculiarity of these conditions suggests that the elevation of Arc/Arg3.1 expression could help to cope with challenging events. In addition, evidence exists showing that Arc/Arg3.1 expression was elevated in the hippocampus and associated with behavioral resilience in rats exhibiting a partial behavioral alteration once exposed to predator stress but not in rats whose behavior was severely disrupted [175]. We can thus infer that increased Arc/Arg3.1 expression may contribute to behavioral resilience, which can be considered another face of coping. However, the possibility exists that when Arc/Arg3.1 expression enhancement persists over time, it might lead to an exaggerated strengthening of synapses, causing synaptic saturation and depotentiation, eventually interfering with physiological neuroplasticity. In addition, we cannot rule out the possibility that Arc/Arg3.1 elevation may instead be an index of inability/failure to adapt to unexpected situations: in line with this possibility, we found that the antidepressant agomelatine is able to normalize stress-induced increase of Arc/Arg3.1 expression [123].

The modulation of the expression of this versatile gene could also be used for different, practical purposes. In fact, we have shown that acute modulation of Arc/Arg3.1 levels in rodents may help to distinguish between the FGA or SGA profile of AP drugs. Therefore, it can be used as a predictive tool to get an idea to which class of antipsychotics a given drug belongs, an add-on value not based on the widely used high/low receptor affinity toward D_2_/5HT_2A_ receptors or on rapid/slow dissociation from dopaminergic D_2_ receptors. In addition, the versatility of this effector gene is further demonstrated by the evidence that the modulation of Arc/Arg3.1 expression can be used to distinguish the brain regions activated following acute or chronic AP treatment. In fact, it appears that repeated treatments de-recruit the brain regions that are activated following acute exposure to drugs, shifting their action toward the frontal cortex rather than the striatum, thus confirming that Arc/Arg3.1 mRNA levels appear to be regulated preferentially at striatal level following acute administration of Aps, whereas the PFC is more likely to be the target site of repeated treatments and this essentially reflects the action of APs in terms of activation/ deactivation of brain regions.

To sum up, although it is difficult to have a clear mechanism that defines the role of Arc/Arg3.1 in the mechanism of action of APs, ADs or psychostimulants, it appears that the modulation of Arc/Arg3.1 could represent a crossroads with structural and important functions in the action of these compounds. Accordingly, modulating Arc/Arg3.1 levels may be an option for the treatment, at least, of some symptoms of psychiatric disorders while taking into account the notion that Arc/Arg3.1 is only a piece of the complex puzzle regulating these multifaceted disorders.

## Figures and Tables

**Table 1:** Summary of the effects produced by single or repeated administration of antipsychotic, antidepressant drugs, and psychostimulants on Arc/Arg3.1 expression.

**Treatment**	**Drugs**	**Species/Sex**	**Dose/Route**	**Arc/Arg3.1 Modulation**	**References**
**Antipsychotics**					
Single treatment	Quinpirole	Male Sprague-Dawley rats	1 mg/kg, i.p.	↓ STR No changes in PFC	[24]
	SCH 23390		1 mg/kg, i.p.	↓ STR, PFC	
	SKF 81293		3 mg/kg, i.p.	↑ STR, PFC	
	Pridopidine	Male Sprague-Dawley rats	3.5 mg/kg, i.p. 10.5 mg/kg, i.p. 32 mg/kg, i.p.	No changes in STR, FC ↑ FC – No changes in STR ↑ STR, FC	[62]
	Ordopidine		3.5 mg/kg, i.p. 11 mg/kg, i.p. 32 mg/kg, i.p.	↑ STR, No changes in FC ↑ STR, FC ↑ STR, FC	
	Haloperidol	Male Sprague-Dawley rats	1 mg/kg, i.p.	↑ STR, ↓ FC	[54]
	Olanzapine		2 mg/kg, i.p.	↑ STR, ↓ FC	
	Quietapine		10 mg/kg, i.p.	No changes in STR	
	Raclopride		2 mg/kg, i.p.	↓ STR, No changes in FC	
	Quinpirole		1 mg/kg, i.p.	↓ STR, No changes in FC	
	Amisulpride	Male Sprague-Dawley rats	10 mg/kg, i.p. 35 mg/kg, i.p.	No changes in dmCP, vmCP, dlCP, vlCP, AC, M2, M1, SS, I, core, shell ↑ dmCP, vmCP No changes in dlCP, vlCP, AC, M2, M1, SS, I, core, shell	[69]
	Haloperidol		0.8 mg/kg, i.p.	↑ dmCP, vmCP, dlCP, vlCP No changes in AC, M2, M1, SS, I, core, shell	
	Asenapine	Male Sprague-Dawley rats	0.05 mg/kg, i.p. 0.1 mg/kg, i.p. 0.3 mg/kg, i.p.	No changes in AC, M2, M1, SS, I, dmCP, vmCP, dlCP, vlCP core, shell ↓ AC, M2, M1 No changes SS, I, dmCP, vmCP, dlCP, vlCP, core, shell ↑ dlCP, vlCP, core No changes in AC, M2, M1, SS, I, dmCP, vmCP, shell	[72]
	Lurasidone	Male Sprague-Dawley rats	1.0 mg/kg, o. s. 3.0 mg/kg, o. s. 10 mg/kg, o. s.	↑ HIP No changes in PFC, STR ↑ HIP No changes in PFC, STR ↑ STR No changes in HIP, STR	[70]
	Sertindole	Male Sprague-Dawley rats	2 mg/kg, i.p.	No changes in dmCP, dlCP, vmCP, vlCP, core, shell	[75]
	SEP-363856	Male Sprague-Dawley rats	1.0 mg/kg, o. s. 3.0 mg/kg, o. s. 10 mg/kg, o. s.	↑ PFC, vHip No changes dHip, STR ↑ PFC No changes vHip, dHip, STR ↑ PFC No changes vHip, dHip, STR	[77]
Repeated treatment	Clozapine	Male Sprague-Dawley rats	20 mg/kg, i.p.	↓ Cg No changes in dlCP, NAc in mPFC	[79]
	Haloperidol		1 mg/kg, i.p.	No changes in dlCP, Nac in mPFC, Cg (24 hrs post-treatment)	
		Male Sprague-Dawley rats	0.25 mg/kg, i.p. 0.5 mg/kg, i.p. 0.8 mg/kg, i.p.	↑ dmCP, dlCP, vmCP, vlCP, core, shell	[80]
		Male Sprague-Dawley rats	1 mg/kg, i.p.	↓ STR, no changes in FC (2 hrs post-treatment) ↓ STR, no changes in FC (24 hrs post-treatment)	[54]
	Olanzapine		2 mg/kg, i.p.	↓ STR, FC (2 hrs post-treatment) ↓ STR, no changes in FC (24 hrs post-treatment)	
	Quetiapine		10 mg/kg, i.p.	↓ in FC, no changes in STR	
	Aripiprazole	Male Sprague-Dawley rats	10 mg/kg, i.p.	↑ PFC, Hip, STR	[87]
	Lurasidone	Male Sprague-Dawley rats	1.0 mg/kg, o. s. 3.0 mg/kg, o. s. 10 mg/kg, o. s.	↑ PFC, STR No changes in Hip ↑ HIP, PFC No changes in STR ↑ HIP, PFC No changes in STR	[70]
		Male Sprague-Dawley rats	10 mg/kg, s. c.	↑ HIP, PFC	[95]
	Blonanserin	Male Sprague-Dawley rats	0.3 mg/kg, o. s. 3 mg/kg, o. s.	↑ vHip, dHip No changes in PFC No changes in vHip, dHip, PFC	[97]
		Male Sprague-Dawley rats	3 mg/kg, o. s. 10 mg/kg, o. s.	↑ dHip ↓ Hyp No changes in PFC, STR, vHip ↓ Hyp No changes in PFC, STR, vHip, dHip	[98]
**Antidepressants**					
Single treatment	Fluoxetine	Male Sprague-Dawley rats	10 mg/kg, p. o.	No changes in CA1, Ca3, DG, PC, FC	[104]
		Male Sprague-Dawley rats	10 mg/kg, i.p.	No changes in Hip	[105]
	Duloxetine	Male Sprague-Dawley rats	10 mg/kg, p. o.	↑ EC, MB ↓ FC No changes in PFC, PC, HIP, STR, HYP	[94]
Repeated treatment	Fluoxetine	Male Sprague-Dawley rats	10 mg/kg, p. o.	↑ in FC No changes in CA1 and PC	[104]
		Male Sprague-Dawley rats	10 mg/kg, i.p.	↑ in Hip	[105]
	Escitalopram	Male Flinders Resistant line (FRL) rats	340-410 mg/kg, pellets	↑ in CA1, DG No changes in Amy, Phr, Scx	[102]
	Nortriptyline	Male Flinders Resistant line (FRL) rats Female C57BL/6 mice	330 mg/kg, pellets	No changes in CA1, DG, AMY, PHR, SCX	
	Vortioxetine		600 mg/kg of food	↑ FC	[112]
	Duloxetine	Male Sprague-Dawley rats	10 mg/kg, p. o.	↑ EC, FC, PFC, MB ↓ PC, STR No changes in HIP, HYP	[94]
Repeated treatment	Agomelatine	Male Sprague-Dawley rats	40 mg/kg, i.p.	↑ HIP No changes in PFC	[122]
	Venlafaxine	Male Sprague-Dawley rats	10 mg/kg, i.p.	↑ HIP ↓ PFC	[122]
		Male Sprague-Dawley rats	10 mg/kg, i.p.	↑ Cg, PC No changes in HIP	[118]
**Psychostimulants**					
Single treatment	Cocaine	Male Sprague-Dawley rats	20 mg/kg, i.p.	↓ mPFC	[136]
		C57BL/6 mice	2.5 mg/kg, i.p.	↑ dm, shell	[138]
		Male Sprague-Dawley rats	10 mg/kg, i.p.	↑ mPFC	[145]
	MDPV	Male CD-1 mice	0.001 mg/kg, i.p. 0.1 mg/kg, i.p. 1 mg/kg, i.p. 10 mg/kg, i.p.	↑ STR, Hip, Frontal lobe	[163]
	aPVP	Male CD-1 mice	0.001 mg/kg, i.p. 0.1 mg/kg, i.p. 1 mg/kg, i.p. 10 mg/kg, i.p.	↑ Frontal lobe No changes in STR, Hip	
	Amphetamine	Male Sprague-Dawley rats	0.5 mg/kg, i.p.	↑ mPFC, core, dm, dl, ObCx No changes shell, vm	[148]
		Male C57BL/6	10 mL/kg, i.p.	↑ STR, NAc	[165]
		Male C57BL/6	2.5 mg/kg, i.p.	↑ F, mPFC, Hipp	[161]
		Male Sprague-Dawley rats	0.5 mg/kg, i.p.	↓ CA1, CA3, DG, ParCx, ↑ dmCP No changes in Cg, Obx (juvenile) ↑ CA1, ParCx, Cg, Obx, dmCP ↓ DG No changes in CA3 (adult)	[166]
	Methylphenidate	Male Sprague-Dawley rats	2 mg/kg, i.p.	↓ CA1, CA3, DG, ↑ Cg, dmCP No changes in ParCx, Obx (juvenile) ↑ CA1, ParCx, Cg, Obx, dmCP ↓ DG No changes in CA3 (adult)	
		Male Sprague-Dawley rats	4 mg/kg, i.p.	↑ Cg, Obx	[167]
		Male Sprague-Dawley rats	2 mg/kg, s.c. 10 mg/kg, s.c.	↑ STR, CFr ↑ STR, CFr	[168]
Repeated treatment	Cocaine	Male Sprague-Dawley rats	20 mg/kg, s.c.	↓ ILc, PLc	[139]
		Male Sprague-Dawley rats	20 mg/kg, s.c.	↑ mPFC	[154]
		Male Sprague-Dawley rats	0.25 mg/0.1 mL/infusion	↑ mPFC	[143]
		Male CD-1 mice	3 ml/kg, i.p.	↑ NAc, dHip, vhip	[156]
	Methylphenidate	Male Sprague-Dawley rats	2 mg/kg, s.c. 10 mg/kg, s.c.	↑ CFr, No changes in STR ↑ CFr, STR	[168]

**Abbreviations:** AC, Cingulate cortex; Amy: Amygdala; CFr: Cingulate/frontal cortex; Cg: Cingulate; DG: Dentate gyrus; dlCP: Dorsolateral Caudato putamen; dmCP: Dorsomedial Caudato putamen; EC: Enthorynal cortex; FC: Frontal cortex; HIP: Hippocampus; HYP: Hypothalamus; I: Insular cotex; ILc: Infralimbic cortex; M1: Motor cortex; M2: Medial Agranular cortex; MB: Midbrain; mPFC: Medial prefrontal cortex; ObCx: Orbitofrontal cortex; ParCx: Parietal cortex; PC: Parietal cortex; Phr: Parahippocampal region; PLc: Prelimbic cortex; Scx: Primary sensory cortex; SS: Somatosensory cortex; STR: Striatum; vHIP: Ventral hippocampus; vlCP: Ventrolateral Caudato putamen; vmCP: Ventromedial Caudato putamen.

## LIST OF ABBREVIATIONS

AD: Antidepressant
AP: Antipsychotic
CNS: Central Nervous System
FSL: Flinders Sensitive Line
IEGs: Immediate Early Genes
NAc: Nucleus Accumbens
NOR: Novel Object Recognition
PFC: Prefrontal Cortex
SNP: Single Nucleotide Polymorphisms
